# A Wide-Band Antenna with Circular Polarization Utilizing a U-Shaped Radiator and Parasitic Strip for Wireless Communications

**DOI:** 10.3390/mi14071308

**Published:** 2023-06-26

**Authors:** Basma M. Yousef, Allam M. Ameen, Meshari D. Alanazi, Maheswar Rajagopal, Ahmed A. Ibrahim

**Affiliations:** 1 Communications Department, Delta Higher Institute for Engineering and Technology, Mansoura 35681, Egypt; 2 Microstrip Department, Electronics Research Institute, New Nozha, Cairo 11843, Egypt; 3 Electrical Engineering Department, College of Engineering, Jouf University, Sakaka 72388, Al Jouf, Saudi Arabia; 4 Department of Electronics and Communication Engineering Centre for IoT and AI (CITI), KPR Institute of Engineering and Technology, Coimbatore 641407, India; 5 Electronics and Communications Engineering Department, Faculty of Engineering, Minia University, Minia 61519, Egypt

**Keywords:** circular polarization (CP), compact antenna, UWB, AR, wireless systems

## Abstract

A circularly polarized (CP) and wide-band monopole antenna with a miniaturized size is suggested in this study. The suggested structure is composed of a U-shaped radiator on the front side, a partial ground plane with two rectangle slots, and a quadrilateral-shaped parasitic strip on the back side of the FR4 substrate. A wide-band operation with *S*_11_ ≤ −10 dB was achieved by regulating the radiator and the partial ground that was placed on the second side of the antenna substrate. The CP was achieved when excited two modes with the same amplitude and a 90° phase difference. This could be generated by regulating the slots’ dimensions in the ground plane. Moreover, a quadrilateral-shaped parasitic strip placed on the second side with the partial ground was utilized to extend the 3 dB axial ratio (AR) bandwidth. The suggested structure is simulated, prototyped, and measured to confirm the desired requirements with a total size of 30 × 32 mm^2^ (0.4 × 0.42 λ_0_ at 4 GHz). The tested outcomes have a bandwidth of *S*_11_ ≤ −10 dB (81.25%) (5.2 GHz, 3.8–9 GHz) and a 3 dB axial ratio (AR) bandwidth (30.7%) (1.63 GHz, 4.48–6.11 GHz). The antenna’s different parameters are discussed, which recommend the suggested antenna to be used in UWB, sub 6 GHz, and WLAN wireless applications.

## 1. Introduction

Recently, wireless communication systems of a small and compact size that operate in a wide-band operation need compact and wide-band antennas that can be easily integrated with them [1,2]. CP antennas have several benefits, such as better mobility, orientation flexibility between the transmitting and receiving ends, decreasing multipath interference, and polarization mismatch [3,4,5]. Two orthogonal modes with equal magnitudes and a 90° phase shift must be generated to produce the CP operation [1].

In modern wireless systems, for example, wide-band circular polarization (CP) antennas, radio frequency identification (RFID), and global positioning systems (GPSs) are recommended and utilized. Recently, wireless systems have been operated at different frequency bands. Therefore, wide-band CP antennas are considered a good choice to reduce the system’s complexity and price [6,7,8]. Microstrip [9,10,11,12], dielectric resonator (DRAs) [7,8], slot [13,14], and monopole antennas [2,3,4,5,15,16,17,18,19,20,21,22,23,24] are used to achieve CP behavior.

Microstrip antennas have several advantages, such as low price, simple design, small size, ease of integration, and simplicity in CP realization. Therefore, the microstrip antenna is used in several communication systems. However, conventional CP antennas are produced with a small 3 dB axial ratio with a large size, which cannot be suitable for wide-band systems [9,10]. For this reason, monopole antennas can be used to produce a wide-band CP operation. The monopole antennas can be fabricated easily and have a small size, low price, stable radiation patterns, wideband bandwidth, and CP operation. Therefore, recently, researchers have extensively studied them [2,4,5]. To enhance the 3 dB AR bandwidth and preserve the antenna compactness, a monopole antenna can be utilized [2,3,4,5,15,16,17,18,19,20,21,22,23,24].

In [2], a parasitic G-shaped strip is added to a C-shaped monopole antenna to generate a CP operation. The antenna is operated from 3.92 to 7.52 GHz (62.9%). The FR4 substrate at 1.6 mm is utilized in the design. The antenna had a size of 30 × 32 mm^2^ and peak gain of 3.6 dB. Additionally, the ground plane is modified to increase the 3 dB AR bandwidth to 53.92% from 4.28 to 7.44 GHz. The antenna has a simple structure with a wide-band 3 dB AR bandwidth and is utilized for WLAN communication.

The UWB CP operation generated using a sequential phase feed is investigated in [3]. It is composed of a monopole antenna with a circular arc-shaped radiator on the top of the RO4003 substrate with a 0.813 mm thickness and a quasi-elliptical strip connected to the partial ground on the other side. The antenna is operated from 1.8 to 7.3 GHz (120.9%). The antenna has a size of 120 × 120 mm^2^ and a peak gain of 11.2 dB. Additionally, the antenna has a 3 dB AR bandwidth from 1.5 to 7.5 GHz (133.33%). While the AR in [3] has a UWB operation, the antenna has a larger size and complex design.

In [4], a broadband antenna with a C-shaped monopole patch on the top side and an open-loop rectangular resonator and modified ground on the back side is investigated to extend the 3 dB AR to 65.2% from 4.28 to 8.42 GHz. A compact broadband antenna with a circular C-shaped patch, an improved ground plane, and a large size to extend the 3 dB AR are investigated in [5]. The antenna is operated from 2.25 to 7.35 GHz (106.3%). The FR4 substrate of 1.6 mm is utilized in the design. The antenna has a size of 49 × 55 mm^2^ and a peak gain of 6.55 dB. Additionally, the ground plane is modified to increase the 3 dB AR bandwidth to 104.7% from 2.05 to 6.55 GHz. The antenna has a simple structure with a wide-band 3 dB AR bandwidth and is utilized for WLAN communication; however, it has a large size.

A microstrip antenna utilizing a fractal-defected ground structure is discussed in [9]. The antenna is worked from 1.55 to 1.58 GHz (1.9%). A substrate of a dielectric constant of 10 and 3.88 mm thicknesses is utilized in the design. The antenna has a size of 45 × 45 mm^2^ and a peak gain of 2.2 dB. The antenna has a simple structure with a narrow 3 dB AR from 1.572 to 1.578 GHz (0.4%). The antenna has a narrow band of 3 dB AR bandwidth with a large size. A 3D with complex structure microstrip antenna is introduced in [10]. An L-shaped slot is etched in the patch. The antenna is operated around 1.9 (27.2%). A substrate of a dielectric constant of 2.7 and 9 mm thicknesses is utilized in the design. The antenna has a size of 80 × 80 mm^2^ and a peak gain of 4.3 dB. The antenna has a 3 dB AR bandwidth of 3%. The antenna has a complex structure with a large size.

In [12], a microstrip antenna with an H-shaped and reactive impedance surface (RIS) is investigated. The antenna is operated from 4.64 to 7.3 GHz (44.5%). The RO4003 substrate with 5.88 mm is utilized in the design. The antenna has a size of 32 × 32 mm^2^. The antenna has a peak gain of 7.2 dB. Additionally, the antenna has a 3 dB AR bandwidth of 27.5% from 4.55 to 6 GHz. The antenna has a complex structure and it is utilized for WLAN communication. A slot antenna with a CPW feed is introduced in [13]. The antenna is operated from 1.78 to 5.64 GHz (104%). The 1.66 mm FR4 substrate is utilized in the design. The antenna has a size of 54 × 54 mm^2^ and a peak gain of 3.8 dB. Additionally, the antenna has a 3 dB AR bandwidth of 58.6% from 2.85 to 5.21 GHz. The antenna has a simple structure with a large size and it is utilized for WLAN communication.

Moreover, some modifications are utilized in the monopole antenna to increase the 3 dB AR bandwidth. Lumped capacitors are used in [15]. An inverted U-shaped monopole antenna attached to lumped capacitors is used to generate the CP operation. The antenna is operated from 3.75 to 7 GHz (60.5%). A substrate of a dielectric constant of 2.2 and 1.52 mm thicknesses is utilized in the design. The antenna has a size of 52 × 55 mm^2^ and a peak gain of 4.8 dB. Additionally, it has a 3 dB AR bandwidth of 4% from 4.25 to 5.95 GHz. The antenna has a simple structure with a large size and works for wireless communication. Parasitic strips are investigated in [16,18]. A Y-shaped monopole antenna is suggested in [19]. The antenna is operated from 2.25 to 2.35 GHz (28.6%). A substrate of a dielectric constant of 2.2 and 3.1 mm thicknesses is utilized in the design. The antenna has a size of 50 × 55 mm^2^ and a peak gain of 2.9 dB. Additionally, it has a 3 dB AR bandwidth of 4% from 2.25 to 2.35 GHz. The antenna has a simple structure with a large size and works for satellite communication.

A slot antenna with an L-shape is suggested in [22]. An L-shaped monopole slot antenna with a C-shaped feed is utilized to generate the CP operation. The antenna is operated from 1.48 to 1.93 GHz (30%). The 0.8 mm FR4 substrate is utilized in the design. The antenna has a size of 70 × 70 mm^2^ and a peak gain of 2.45 dB. Additionally, it has a 3 dB AR bandwidth of 32% from 1.42 to 1.97 GHz. The antenna has a simple structure with a large size and works for satellite communication. A parasitic open-loop resonator is added to a rectangular monopole antenna to generate the CP operation. The antenna is operated from 1.48 to 4.24 GHz (96.5%). The 1 mm FR4 substrate is utilized in the design. The antenna has a size of 50 × 55 mm^2^ and a peak gain of 3.5 dB. Additionally, the ground plane is modified to increase the 3 dB AR bandwidth to 63.3% from 2.05 to 3.95 GHz. The antenna has a simple structure with a wideband 3 dB AR bandwidth and a large size.

A wide-band CP antenna is introduced in this work. A U-shaped radiator on the front side, a partial ground plane with two rectangle slots, and a quadrilateral-shaped parasitic strip on the back side are utilized to produce the CP feature. The CP is achieved when we excited two modes with the same amplitude and a 90° phase difference. This can be generated by regulating the slot’s dimensions and adding a quadrilateral-shaped parasitic strip to the ground plane. The tested outcomes have a bandwidth of S_11_ ≤ −10 dB (81.25%) (5.2 GHz, 3.8–9 GHz) and a 3 dB axial ratio (AR) bandwidth (30.7%) (1.63 GHz, 4.48–6.11 GHz). The designed antenna keeps the same features as a common turnstile antenna, such as simplicity, low cost, compact, low profile, and broadband CP antenna. All simulated results are extracted utilizing CST software. The suggested antenna can be used in UWB, sub 6 GHz, and WLAN wireless applications.

The paper is organized as follows: (I) The literature review is introduced. (II) The configuration of the antenna is investigated. (III) The simulation and measurement outcomes are provided. (IV) The conclusion is presented.

## 2. Proposed Antenna Structure

### 2.1. Antenna Design and Configuration

**Figure 1:**
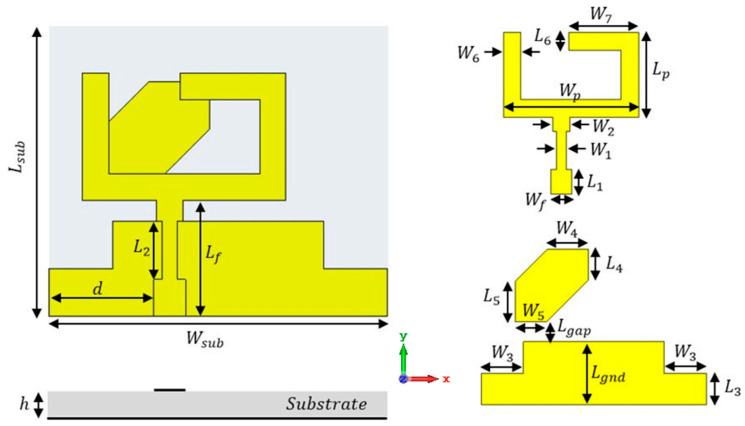
The antenna’s complete geometrical configuration.

The proposed wide-band CP monopole antenna with the complete geometrical configuration is shown in Figure 1. FR4 with a total size of 32 × 30 × 1.6 mm^3^ and εr = 4.4, tan δ = 0.025 was used as a substrate. The suggested antenna was connected to a 50 Ω microstrip feed line with an optimal position to enhance the matching of the antenna. An L-shaped connected to a C-shaped mirror to compose a suggested U-shaped radiator was added to the front side. Moreover, a partial ground plane with two rectangle slots and a quadrilateral-shaped parasitic strip was added to the back side of the substrate. The two rectangle slots were cut from the edge of the ground plane to achieve CP modes at high frequencies. The wide CP generation was achieved by adding a quadrilateral strip to the bottom of the substrate, as illustrated in Figure 1. It was used to create a CP mode at a lower frequency that, in turn, improved the AR over the desired frequency. Table 1 presents the dimensions of the antenna.

**Figure 2:**
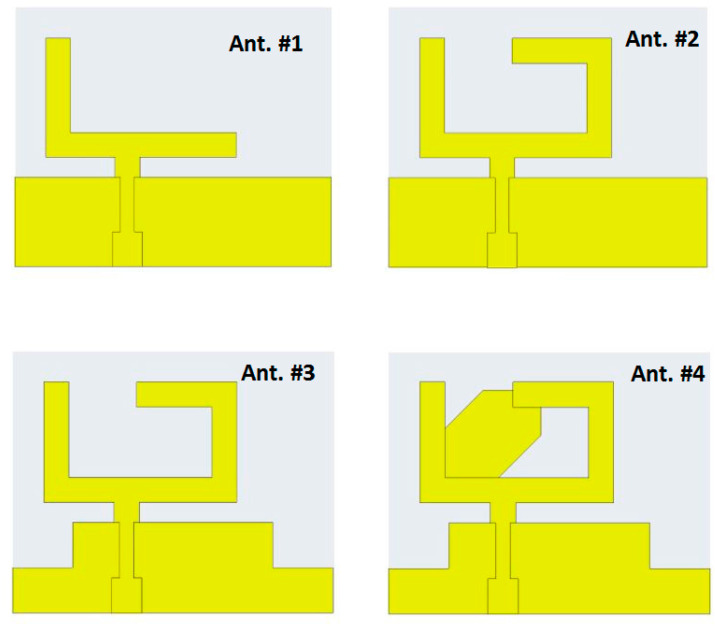
The antenna design improvement procedures.

**Figure 3:**
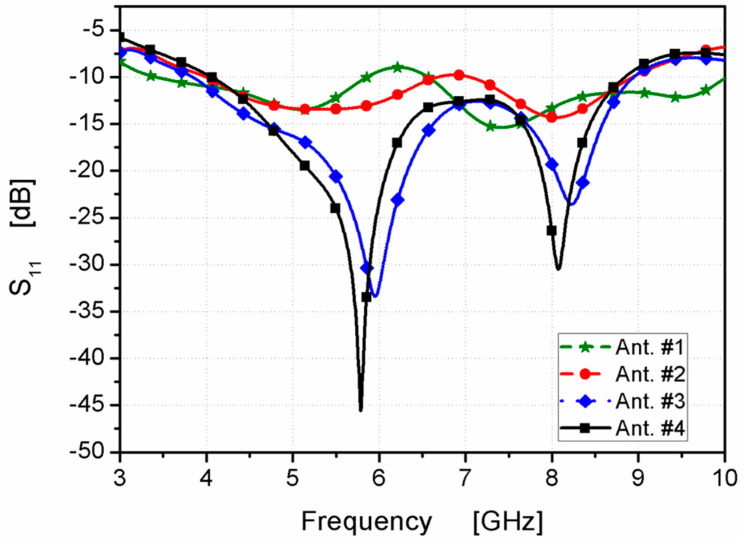
S_11_ outcomes of the designed four antennas.

**Figure 4:**
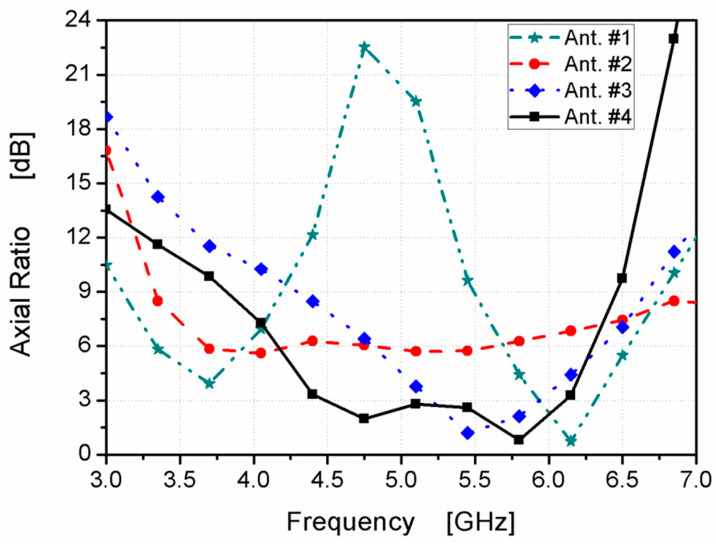
ARs of the designed four antennas.

The suggested structure was passed through four stages to achieve the desired final design. The four-step antenna design procedure is illustrated in Figure 2. In Ant. #1, an L-shaped radiator on the front side with the partial ground on the bottom was designed, as shown in Figure 2. As displayed in Figure 3 (the green curve), the antenna is operated at dual-wide-band ranges. The first started from 3.5 to 5.9 GHz and the second worked from 6.5 up to 10 GHz. To extend the impedance bandwidth to cover the two ranges from 4 up to 9 GHz, as shown in Figure 3 (the red curve), the mirror of a C-shaped radiator was connected to an L-shape to achieve the U-shaped radiator, as shown in Figure 2 (Ant. #2). From the two steps, the desired wide-band operating bandwidth can be achieved; however, the CP behavior is not generated, which is related to AR, as shown in Figure 4. Therefore, to achieve the CP feature, a rectangular part was cut from the two edges of the ground plane as Ant. #3 was introduced to improve the antenna matching, as shown in Figure 2 and Figure 3 (blue curve). Moreover, a CP mode was excited at 5.5 GHz, as shown in Figure 4. The 3 dB AR was operated from 5.2 to 6 GHz.

The CP radiation can be obtained by achieving two orthogonal modes with equal amplitudes and a 90° phase difference. However, the conventional monopole antenna, as shown in Figure 2 (Ant. #1, Ant. #2), has little radiation in the horizontal direction. In addition, the horizontal currents in the ground plane moved in opposite directions; therefore, the horizontal current was annulled, which decayed the horizontal polarization. Therefore, linear vertical polarization was generated, as illustrated in Figure 4. Thus, by modifying the ground plane and adding the parasitic strip, as shown in Figure 2 (Ant. #3, Ant. #4), orthogonal horizontal and vertical currents were produced. Moreover, the direction of the current distribution in the ground plane changed, generating CP and increasing the AR value, which tended to increase the bandwidth of the axial ratio, as shown in Figure 4.

Finally, in Ant. #4, the back side of the structure was added with a quadrilateral shape, as shown in Figure 2, to enhance the 3 dB AR band. A quadrilateral-shaped parasitic strip was utilized to balance the electric-field magnitudes of both vertical and horizontal components to make them achieve the same value with a 90° phase difference between them for the CP generation. By adding the parasitic strip, the path of the electric current was increased in the antenna that shifted the frequency band. Furthermore, the CP mode was generated at a lower frequency band from 4.48 to 6.11 GHz, as shown in Figure 4.

**Figure 5:**
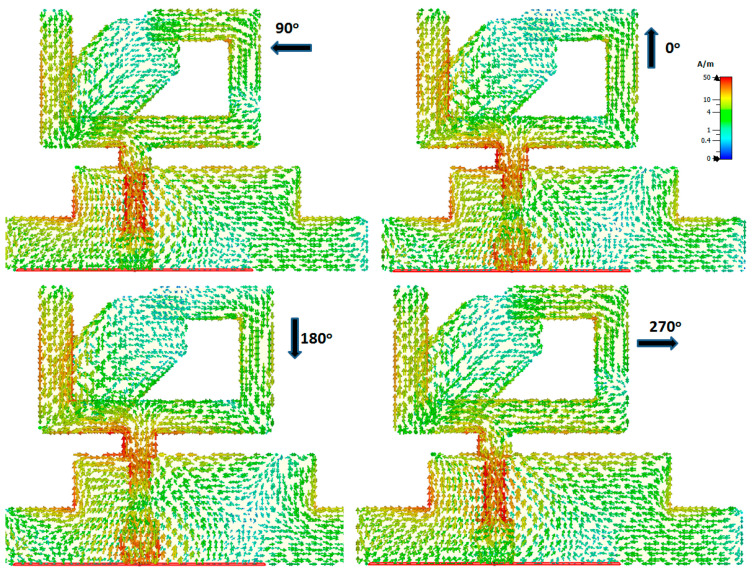
Surface current density at 5.8 GHz during different orthogonal phases.

The CP generation behavior could be understood by displaying the antenna distribution current at different orthogonal phases at 5.8 GHz, as presented in Figure 5. It is shown that at 0° the current was generated along the positive Y-direction. At the 90° phase, it was radiated along the negative X-direction, while at 180° and 270°, the currents were generated in the negative Y- and positive X-directions, respectively. Moreover, we concluded that the current rotated in a counter-clockwise direction.

### 2.2. Analysis and Parametric Study

**Figure 6:**
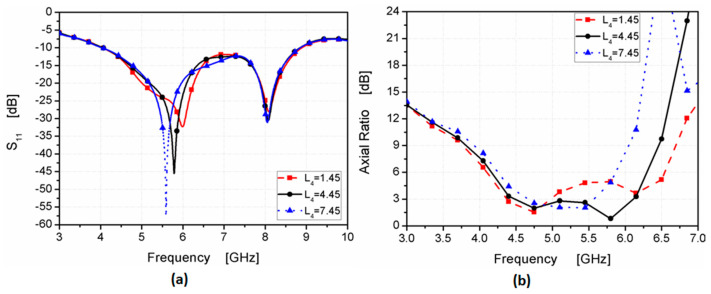
The L4 influence on antenna performance (**a**) *S*_11_; (**b**) AR.

From the previous section, it can be noticed that the dimension of the quadrilateral-shaped strip can affect the 3 dB AR. Therefore, a parametric study was utilized to show its effect. Figure 6 shows the effect of *L*_4_ on the antenna’s performance. By increasing the length of *L*_4_ from 1.45 to 7.45 mm, the antenna was operated from 4 to 9 GHz with a good impedance bandwidth as shown in Figure 6a, while the 3 dB AR bandwidth decreased as illustrated in Figure 6b. When *L*_4_ = 1.45 mm, the 3 dB AR bandwidth was extended from 4.48 to 4.8 GHz. Additionally, when *L*_4_ = 4.45 mm, the 3 dB AR bandwidth was extended from 4.48 to 6.11 GHz. Finally; when *L*_4_ = 7.45 mm, the 3 dB AR bandwidth was extended from 4.7 to 5.5 GHz. The length of *L*_4_ was chosen to be 4.45 mm.

**Figure 7:**
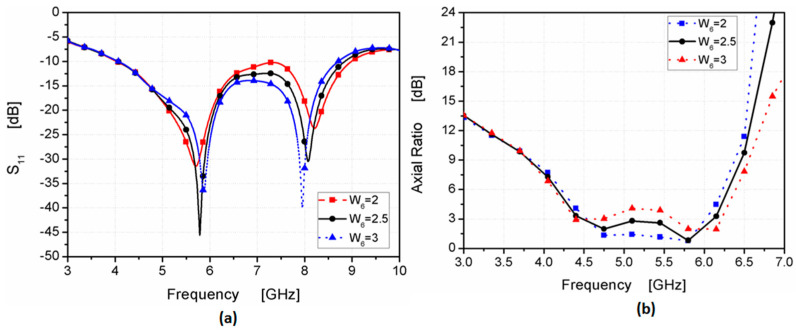
The W6 influence on antenna performance (**a**) *S*_11_; (**b**) AR.

Furthermore, Figure 7 shows the W6 effect on the antenna’s performance. By increasing the length of W6 from 2 to 3 mm, while keeping *L*_4_ = 4.45, the antenna was operated from 4 to 9 GHz with a good impedance bandwidth as illustrated in Figure 7a, while the 3 dB AR bandwidth decreased as shown in Figure 7b. When *W*_6_ = 2 mm, the 3 dB AR bandwidth was extended from 4.48 to 6 GHz. Moreover, when *W*_6_ = 2.5 mm, the 3 dB AR bandwidth was extended from 4.48 to 6.11 GHz. Finally, when *W*_6_ = 3 mm, the 3 dB AR bandwidth was extended from 4.48 to 5 GHz and from 5.6 to 6.2 GHz. The length of W6 was chosen to be 2.5 mm. Finally, by elaborating on the parametric study outcomes, the final dimensions achieved the desired bandwidth from 4 to 9 GHz and satisfied the 3 dB AR bandwidth from 4.48 to 6.11 GHz.

## 3. Experimental Outcomes and Investigations

**Figure 8:**
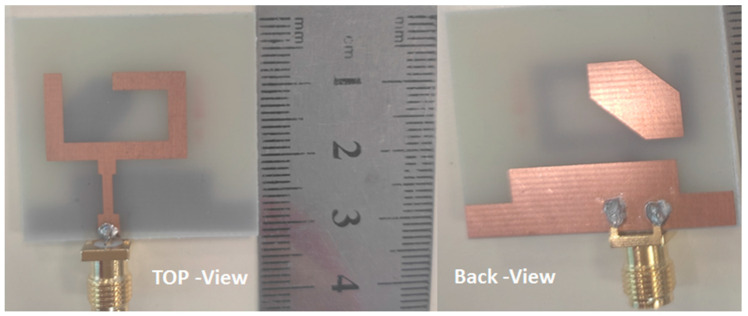
Fabricated prototype layout of suggested antenna.

**Figure 9:**
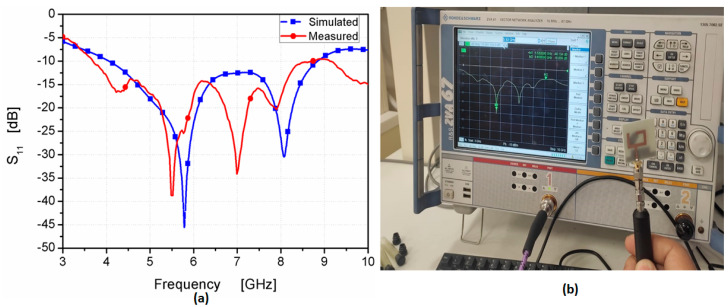
The S_11_ results of the suggested antenna: (**a**) simulated and measured (**b**) VNA screenshot.

The photolithography method was utilized in the fabrication process. Figure 8 shows the prototype layout photo of the suggested antenna in the top and back views. FR4, with a total size of 32 × 30 × 1.6 mm^3^, and εr = 4.4, tan δ = 0.025 were used in the fabrication. Additionally, it was tested using a vector network analyzer (R&S ZVA 67) to show the reflection coefficient *S*_11_, as shown in Figure 9a. The VNA screenshot result of the tested antenna is illustrated in Figure 9b. The tested results show the antenna worked at the frequency band from 3.8–9 GHz (81.25%) with *S*_11_ ≤ −10 dB, while the simulated outcomes illustrate that the antenna is operated from 4 to 9 GHz. The two results display good matching between them, with some slight deviations between them due to the fabrication tolerance and SMA soldering process.

**Figure 10:**
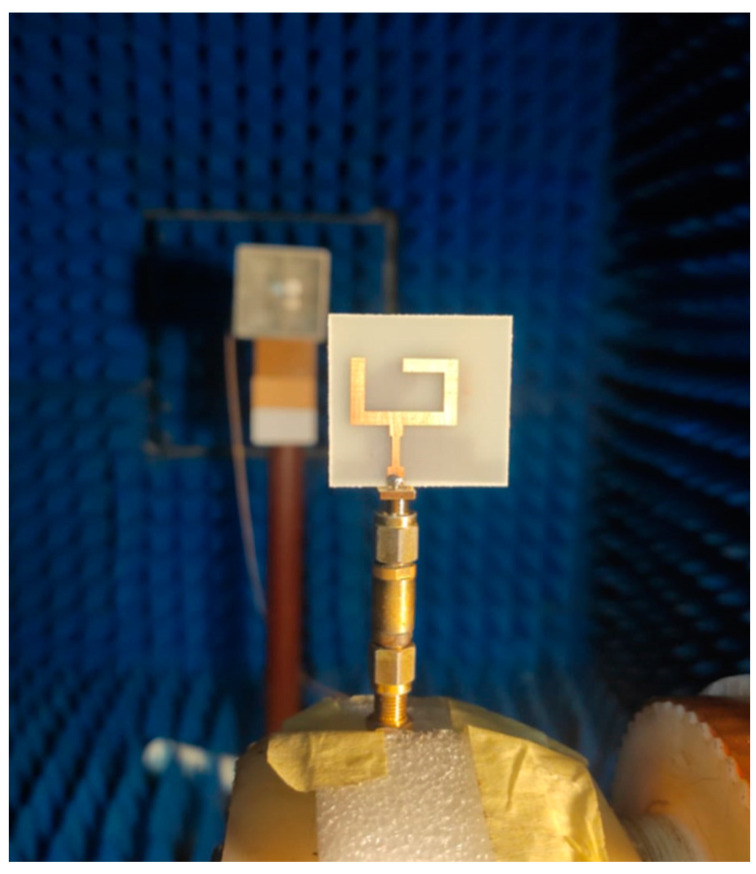
The setup of the radiation pattern measurement.

**Figure 11:**
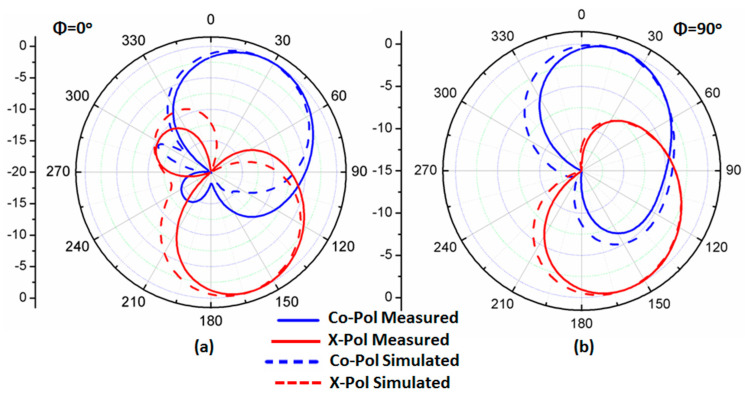
The radiation pattern normalized outcomes at 5.5 GHz (**a**) φ = 0°; (**b**) φ = 90°.

**Figure 12:**
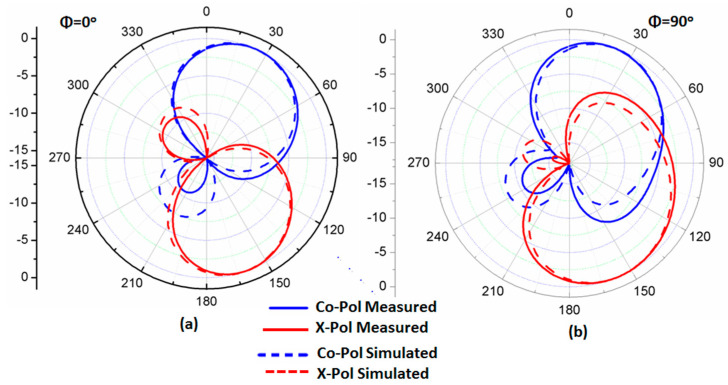
The radiation pattern normalized outcomes at 5.8 GHz (**a**) φ = 0°; (**b**) φ = 90°.

The setup of the far-field results was conducted inside an anechoic chamber, as illustrated in Figure 10. A horn antenna operating at a suitable range of frequency and connected to an RF signal generator was utilized as a transmitter antenna. On the other hand, there was a suggested antenna (antenna under test), which was placed inside the same chamber on a supporter that could rotate 360° in both the elevation and horizontal planes. The antenna was connected to a spectrum analyzer to measure the received signal. Additionally, there was a motion controller that controlled the motion of the antenna. The overall equipment, such as the motion controller, spectrum analyzer, and RF generator, was controlled to be operated synchronously. The antenna rotated around its axis with a certain step and stopped for some seconds; on the other hand, the spectrum analyzer measured the received power at this angle during the stopping time. The suggested antenna was rotated in two planes *xz* (φ = 0°) and *yz* (φ = 90°) planes. The co- and cross-polarization results at 5.5 and 5.8 GHz are shown in Figure 11a,b and Figure 12a,b, respectively. More than a −15 dB difference between the two components was accomplished in both planes.

**Figure 13:**
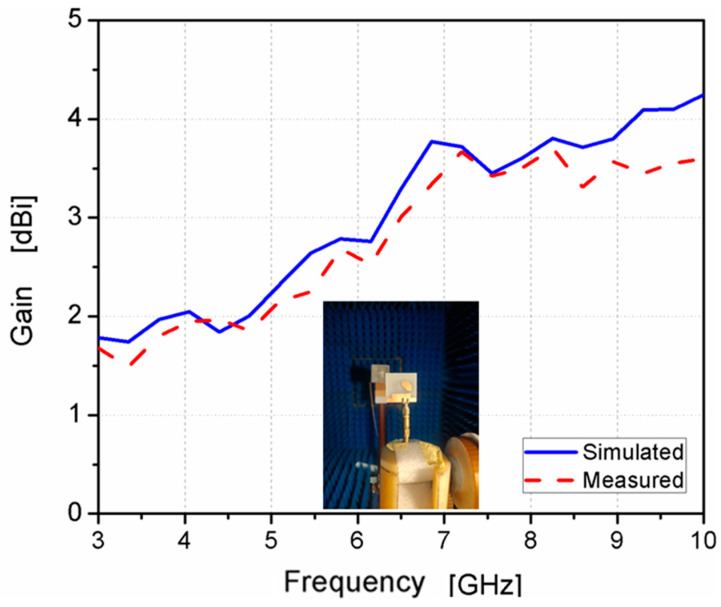
The antenna’s realized gain.

**Figure 14:**
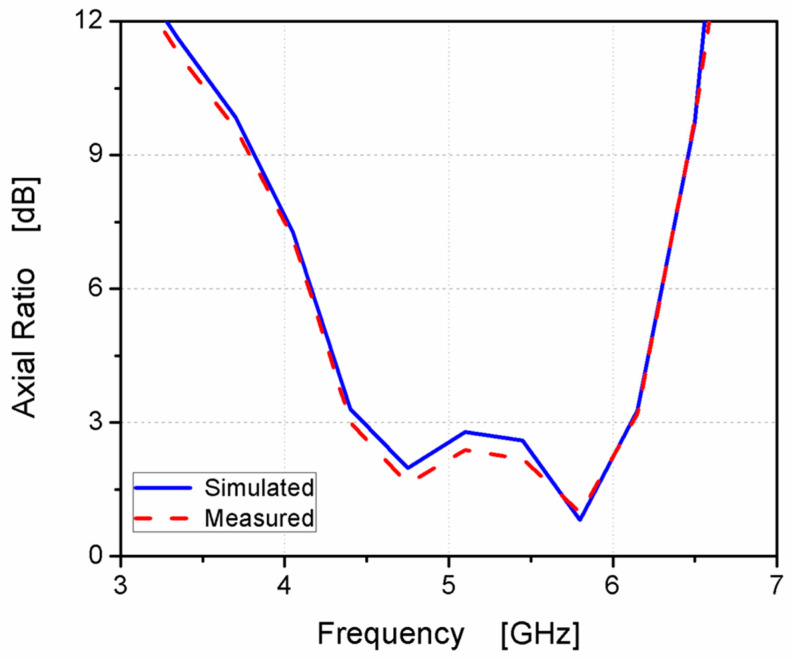
The antenna AR’s measured and simulated outcomes.

The gain of the antenna was measured, as illustrated in Figure 13. It ranged from 1.8 to 3.8 dBi at the designed frequency band with a peak of 3.6 dBi. Moreover, the AR was tested, as displayed in Figure 14, and the achieved 3 dB AR extended from 4.48–6.11 GHz (30.7%) with a reasonable trend between the two outcomes.

Finally, the suggested design was compared with others to evaluate the novelty of the work, as presented in Table 2. The suggested antenna had a more simple design than [3,10,11], a more compact size than [5,9,13,14,15,16,17,18,19,22,23,24,25], a broader bandwidth than [2,9,10,12,15,17,19,21,22,25], and a broader 3 dB AR than [9,10,12,15,19]. Finally, it was deduced that the suggested antenna could be applied in UWB, sub 6 GHz, and WLAN wireless applications.

## 4. Conclusions

A miniaturized size and wide-band CP monopole antenna was suggested, fabricated, and tested. The suggested antenna was 30 mm × 32 mm (0.4 × 0.42 λ_0_ at 4 GHz).

A U-shaped radiator on the front side, a partial ground plane with two rectangle slots, and a quadrilateral-shaped parasitic strip on the back side were utilized to produce the CP feature. The CP was achieved when we excited two modes with the same amplitude and a 90° phase difference. This could be generated by regulating the slot’s dimensions and adding a quadrilateral-shaped parasitic strip to the ground plane. The antenna’s different parameters were discussed and investigated. The tested outcomes had a bandwidth of *S*_11_ ≤ −10 dB 81.25% (5.2 GHz, 3.8–9 GHz) and an AR bandwidth of 30.7% (1.63 GHz, 4.48–6.11 GHz). Based on the achieved outcomes, it can be suggested that the antenna is considered a good choice for several wireless systems, such as UWB, sub 6 GHz, and WLAN applications.

## Figures and Tables

**Table 1:** The antenna dimensions.

Parameter	Length [mm]	Parameter	Length [mm]	Parameter	Length [mm]
$L_{sub}$	30	$L_{3}$	4.5	$L_{g}$	2.8
$L_{p}$	12	$L_{4}$	4.45	h	1.6
$L_{f}$	11	$L_{5}$	5.77	$W_{sub}$	32
$L_{1}$	3.5	$L_{6}$	2.5	$W_{p}$	19.25
$L_{2}$	5.5	$L_{gnd}$	9	$W_{f}$	3
$W_{1}$	1.4	$W_{3}$	6	$W_{5}$	4.45
$W_{2}$	2.5	$W_{4}$	5.77	$W_{6}$	2.5
$W_{7}$	10	d	9.9		

**Table 2:** The suggested design in comparison with other works.

Refs.	Size (mm^2^)	εr/h (mm)	*f*_0_/BW (%)	AR (%)	Gain (dBi)	Complexity
[2]	30 × 32	4.4/1.6	5.72/62.94	53.92	3.6	Simple
[3]	120 × 120	3.55/0.813	4.55/120.9	133.3	11.2	Complex
[5]	49 × 55	4.4/1.5	4.3/106.3	104.7	6.55	Simple
[9]	45 × 45	10/3.18	1.575/1.9	0.4	2.2	Simple
[10]	80 × 80	2.7/9	1.9/27.2	3	4.3	Complex
[11]	90 × 90	3.38/0.8	2/115.2	106.1	7	Complex
[12]	32 × 32	3.38/5.588	5.5/44.5	27.5	7.2	Complex
[13]	54 × 54	2.55/1	4/104	58.6	3.8	Simple
[14]	48 × 48	4.4/1	4.2/114.4	110.5	4.5	Simple
[15]	52 × 55	2.2/1.52	5.4/60.5	30.7	4.8	Simple
[16]	35 × 42	4.4/1.6	3.95/118	104.4	5.2	Simple
[17]	70 × 70	4.7/3.2	2.45/51.4	56.2	2	Simple
[18]	50 × 55	4.4/1	3.4/88	64.7	2.25	Simple
[19]	50 × 55	2.2/3.1	2.45/28.6	4	2.9	Simple
[20]	32 × 39	4.4/1.5	4.9/102	37.5	2.25	Simple
[21]	30 × 30	4.4/1	6.3/55.5	42.6	5.3	Simple
[25]	40 × 40	4.4/1.6	7.9/73.39	58.08	4.22	Simple
[22]	70 × 70	4.3/0.8	1.7/30	32	2.45	Simple
[23]	50 × 50	3.5/1.52	5.8/101	49.8	4.36	Simple
[24]	50 × 55	4.4/1	2.86/96.5	63.3	3.5	Simple
This work	30 × 32	4.4/1.6	6.4/81.25	30.7	3.8	Simple

## Data Availability

The data will be made available at a reasonable request to the corresponding author.
